# Ontogeny‐based immunogens for the induction of V2‐directed HIV broadly neutralizing antibodies

**DOI:** 10.1111/imr.12501

**Published:** 2017-01-30

**Authors:** Penny L. Moore, Jason Gorman, Nicole A. Doria‐Rose, Lynn Morris

**Affiliations:** ^1^ Centre for HIV and STIsNational Institute for Communicable Diseases of the National Health Laboratory ServiceJohannesburgSouth Africa; ^2^ Faculty of Health SciencesUniversity of the WitwatersrandJohannesburgSouth Africa; ^3^ Centre for the AIDS Programme of Research in South Africa (CAPRISA)University of KwaZulu‐NatalDurbanSouth Africa; ^4^ Vaccine Research CenterNational Institute of Allergy and Infectious DiseasesNational Institutes of HealthBethesdaMDUSA

**Keywords:** broadly neutralizing antibodies, HIV, long CDR H3, ontogeny, trimeric immunogens, V2‐apex

## Abstract

The development of a preventative HIV vaccine able to elicit broadly neutralizing antibodies (bNAbs) remains a major challenge. Antibodies that recognize the V2 region at the apex of the HIV envelope trimer are among the most common bNAb specificities during chronic infection and many exhibit remarkable breadth and potency. Understanding the developmental pathway of these antibodies has provided insights into their precursors, and the viral strains that engage them, as well as defined how such antibodies mature to acquire breadth. V2‐apex bNAbs are derived from rare precursors with long anionic CDR H3s that are often deleted in the B cell repertoire. However, longitudinal studies suggest that once engaged, these precursors contain many of the structural elements required for neutralization, and can rapidly acquire breadth through moderate levels of somatic hypermutation in response to emerging viral variants. These commonalities in the precursors and mechanism of neutralization have enabled the identification of viral strains that show enhanced reactivity for V2 precursors from multiple donors, and may form the basis of germline targeting approaches. In parallel, new structural insights into the HIV trimer, the target of these quaternary antibodies, has created invaluable new opportunities for ontogeny‐based immunogens designed to select for rare V2‐bNAb precursors, and drive them toward breadth.

This article is part of a series of reviews covering B cells and Immunity to HIV appearing in Volume 275 of *Immunological Reviews*.

## 1 Introduction

The design of an effective preventative HIV vaccine continues to represent a major public health challenge, with 36 million people currently living with HIV. Despite more than 17 million people now accessing antiretroviral treatment globally and an array of prevention tools, new infections continue to occur at high rates, particularly in parts of southern Africa, with 2 million new infections estimated to occur annually. Many successful vaccines rely on the elicitation of neutralizing antibodies to block viral entry and provide sterilizing protection. However, this is a particular challenge for HIV, which is among the most variable and heavily glycosylated viruses known. Several vaccines have been tested with little or no efficacy, and none of these vaccines have been able to elicit the types of broadly neutralizing antibodies (bNAbs) that will be required to be effective against the enormous global diversity of HIV. Despite these setbacks, there is strong rationale for pursuing bNAbs to prevent HIV infection. Passive immunization of bNAbs isolated from infected donors has long been known to protect non‐human primates from infection [reviewed in 1]. Indeed, a recent study showed that a single injection of bNAbs protected animals against repeated exposure for up to 23 weeks.^2^ Furthermore, studies of HIV infected donors have shown that the human immune system has the capacity to make such bNAbs. These findings, along with the failure of traditional vaccine strategies, has led the field to consider next‐generation vaccine regimens which are based on detailed studies of the ontogeny of bNAbs during infection, a strategy referred to as the B cell lineage approach.^3^ Here, we describe recent virological, immunological, and structural studies supporting and informing this approach, specifically focusing on bNAbs that target the V2 region at the apex of the envelope trimer.

## 2 Why is V2 an attractive bNAb vaccine target?

**Figure 1:**
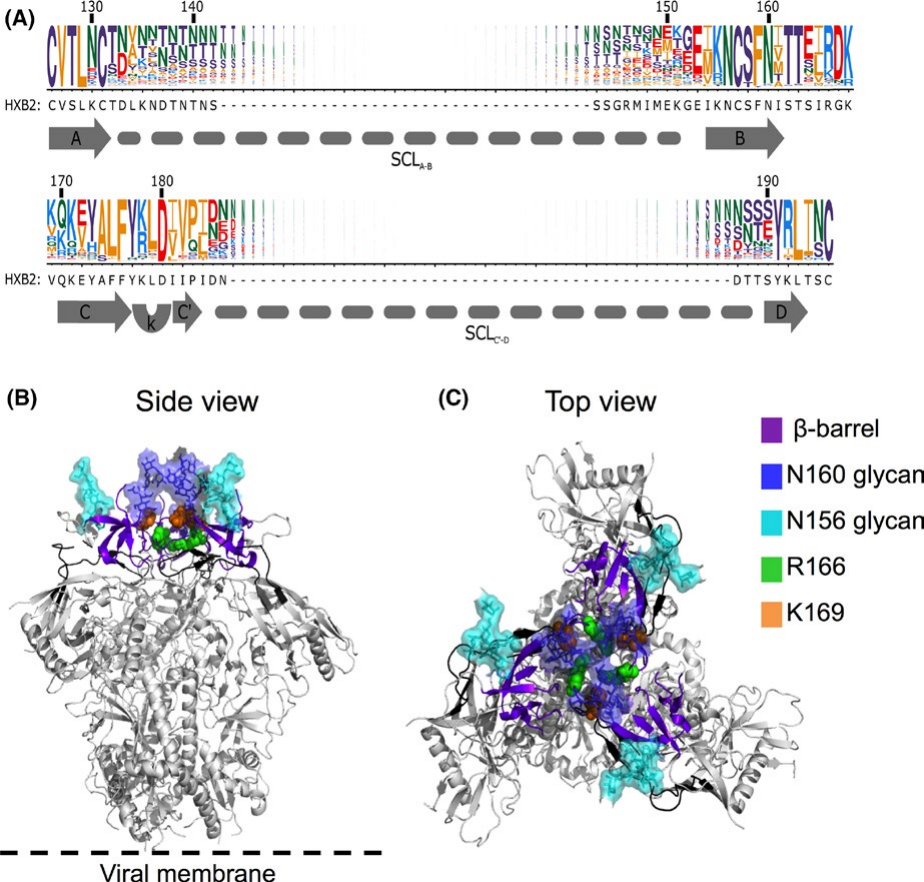
Global alignment and trimeric location of V1V2 within the envelope trimer. (A) Logogram illustrating the amino acid profile of the 2015 version of the LANL premade alignment for Group M V1V2 envelope sequences. The size of each amino acid in the profile indicates prevalence of that amino acid in global sequences. The V1V2 sequence of HXB2 is placed below the profile. Beta strands and strand‐connecting loops (SCL_A__‐B_ and SCL_C__‐D_) are illustrated by arrows and dashed lines respectively. Modified from 58 (B) Side view ribbon representation of the HIV envelope trimer, highlighting the location of the V1V2 domain at the trimer apex. The five anti‐parallel beta strands (beta‐barrel) are shown in purple, while stick and surface representations of the N156 and N160 glycans are shown in cyan and blue respectively. Key amino acid residues at position 166 and 169 are shown as spheres in green and orange respectively. The approximate position of the viral membrane is indicated. (C) Ribbon representation of the trimer showing the view from the angle of approach by V2‐apex bNAbs. PDB ID: 4TVP. N‐linked glycans were modeled using http://www.glycosciences.de/modeling/glyprot/php/main.php

The HIV‐1 envelope (Env) glycoprotein complex, which consists of a heterotrimer of three molecules of gp120 and three molecules of gp41, is responsible for mediating viral entry into host cells, and is the sole target of neutralizing antibodies. The first and second variable regions (V1V2) of gp120 are located at the apex of the envelope trimer, and are highly variable in terms of sequence, glycosylation and length, largely due to mutations and insertions in two regions, in the middle of V1 and toward the C‐terminal end of V2 (Figure 1). In contrast, semi‐conserved regions exist, particularly in the V2 region which is the focus of this review, in strands B and C, including highly conserved glycans at positions 156 and 160 and other fairly conserved residues such as those 166 and 169, which will be described in further detail below. The V1V2 domain is an important contributor to viral entry and neutralization resistance of HIV isolates [reviewed in 4]. Studies of transmission pairs suggest that in many cases infection is mediated by viruses with compact V1V2 regions, which subsequently become longer during the course of infection, suggesting a complex interplay between infectivity and the need for neutralization resistance through V1V2 sequence changes, elongation and glycosylation. The role of the V1V2 region in immune evasion is emphasized by the extreme neutralization sensitivity of V1V2‐deleted viruses.^5^, ^6^, ^7^, ^8^, ^9^ The V1V2 region is itself a frequent target of neutralizing antibodies that drive viral escape mutations within this region.^10^, ^11^, ^12^, ^13^, ^14^, ^15^ In some cases, multiple unrelated B cell lineages target this region, highlighting the immunogenicity of V1V2 during infection.^10^ The high variability in this region results in the majority of these autologous neutralizing responses being strain‐specific and easy for the virus to evade, providing limited insights for HIV vaccine design. However, within this region, the semi‐conserved elements of V2 may also be the target of bNAbs able to recognize diverse circulating viruses, and these are the basis of this review. Thus, while the V1V2 region has long been recognized as a target for antibodies, it was only in 2009 with the isolation of the bNAbs PG9/PG16 that its relevance for vaccine design was truly appreciated.

While almost all infected people develop antibodies to the HIV envelope which have some cross‐neutralizing activity,^16^ only about 20%‐30% of people develop responses that are considered truly broadly neutralizing.^17^, ^18^, ^19^, ^20^ Several factors have been associated with the development of bNAbs during infection, including various measures of germinal center T cell function,^17^, ^19^, ^20^, ^21^, ^22^, ^23^, ^24^, ^25^, ^26^, ^27^ low CD4^+^ counts,^17^, ^18^ HLA and viral subtype.^18^ In addition, several cohort studies have shown that a major contributor to the development of bNAbs is high levels of antigenic stimulation, in the form of high viral loads and duration of infection,^19^, ^20^, ^28^ though there are occasional reports of viral controllers who nonetheless mount bNAb responses.^29^ BNAbs normally emerge only several years after infection, and frequently have unusual features that are not favored by the immune system, including autoreactivity, very long or short CDRs depending on the class of antibodies and high levels of somatic hypermutation (SHM), in some cases >30%.^1^ The association with duration of infection and the high levels of SHM of many HIV bNAbs suggest a long co‐evolutionary pathway, requiring variation in both the virus and the antibody. Indeed, two recent studies suggest that bNAb lineages evolve as quickly as HIV, particularly during the early stages of their development, though these rates later decline.^30^, ^31^ This suggests that even during infection in the context of high levels of viral replication and antigenic stimulation, the development of bNAbs is a difficult pathway. These challenges in developing bNAbs are obviously even greater in the context of vaccination, where antigenic stimulation is normally limited to two or three immunogen exposures, and SHM is normally restricted to about 6%.^32^

Despite these impediments to bNAb development, much of the surface of the HIV trimer is now known to be vulnerable to bNAbs, with many antibody epitopes including the glycans that provide half of the molecular weight of the HIV envelope, and were initially assumed to function solely as a shield for underlying epitopes.^33^, ^34^ These findings come from the isolation of dozens of broadly neutralizing monoclonal antibodies,^35^, ^36^ largely due to the optimization of relatively new technologies for human antibody isolation including antigen‐specific sorting (most recently using trimeric antigens which are particularly important for the quaternary V2‐apex bNAbs^37^, ^38^), B cell culture with micro‐neutralization assays for screening of individual wells, and multiplex RT‐PCR for amplifying immunoglobulin genes from single cells [reviewed in 35, 39, 40]. Characterization of these new bNAbs has enabled the field to define several conserved viral epitopes including the V2‐apex site that is the focus of this review, as well as the N332 glycan supersite, the membrane proximal external region (MPER), the CD4 binding site (CD4bs), and the gp120‐gp41 interface, recently shown to include the fusion peptide.^33^, ^41^ Although many vaccine strategies aim to elicit a polyclonal response that would ideally target multiple conserved epitopes, several approaches are target‐specific. The use of strategies such as minimal epitopes, and have thus resulted in a concerted effort to understand the breadth of antibodies to specific epitopes such as the V2‐apex.

**Figure 2:**
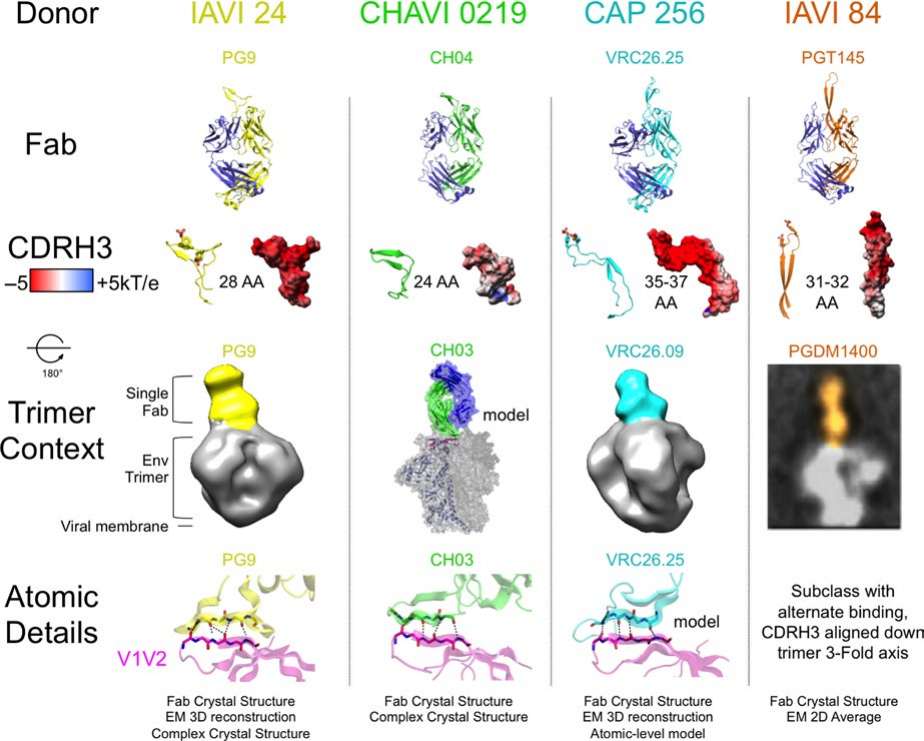
Broadly neutralizing V1V2‐directed antibodies share common structural features. Fabs for prototypical members of each lineage are presented in cartoon representation. Unusually long CDR H3s protrude beyond the framework region (upper row). The CDR H3 regions are shown close up as cartoon with sulfated tyrosines displayed as sticks. Electrostatic charge is shown depicted on the surface to the right of each cartoon representation (second row). Antibodies are shown in the trimer context with 3D EM reconstructions for PG9 and CAP256.09, 2D EM class average for PGDM1400, and an alignment to trimer model for CH03 (row 3). Lineages from each donor show a binding stoichiometry of one fab per trimer. PGDM1400 engages as a steeper angle down the center of the threefold axis rather than off‐center as observed for other V1V2 bNAbs. Crystal structures of PG9 and CH03 highlight atomic‐level detail of antibody binding. CAP256.25 atomic interactions are modeled based on EM, MD, HDX and paratope mapping. PGT145/PGDM1400 atomic level details are unknown but data suggest binding differs from other members of this extended class (bottom row)

Antibodies to the V2‐apex may be especially attractive from a vaccine perspective because they are among the most prevalent of broadly neutralizing responses, with mapping studies in several cohorts of infected donors showing that these account for up to a third of bNAb responses.^17^, ^18^, ^42^, ^43^, ^44^ This indicates that the immune system of many individuals is amenable to the development of these specificities. Furthermore, monoclonal antibodies (mAbs) to this epitope have been isolated from several donors (PG9/PG16 from donor IAVI24, the CH01‐04 lineage from donor CH0219, the PGT145/PGDM1400 lineage from IAVI84, and the CAP256‐VRC26 lineage from CAP256) enabling a comprehensive understanding of their features and neutralization capacity^37^, ^38^, ^45^, ^46^, ^47^ (Table 1 and Figure 2). These mAbs all have structural properties in common, such as an unusually long CDR H3 loop that is generally highly anionic. This negative CDR H3 charge is provided both by an abundance of aspartate and glutamate residues, and by sulfation of tyrosines, and facilitates recognition of the positively charged V2 epitope through a shared mode of recognition, described below (Figure 2).

**Table 1:** Broadly neutralizing V2‐apex antibody characteristics

Donor	Antibody lineage	IgG chain	V gene	J gene	V mutation (%nt)	CDR H3 length	CDR H3 tyrosine sulfation	N160 glycan dependence	Breadth	Reference
IAVI24	PG9; PG16	Heavy	3‐33*05	6*03	12‐15	28	Yes	Yes	75%‐81%	Walker et al. 46
		Light	L2‐14*01	L3*02	8‐12					
CAP256	CAP256‐VRC26.01‐33	Heavy	3‐30*18	3*02	4.2‐18	35‐37	Yes	Partial (potency‐dependent)	2%‐63%	Doria‐Rose et al. 47
		Light	L1‐51*02	L7*01	2.5‐15					
CH0219	CH01‐CH04	Heavy	3‐20*01	2*01	14‐16	24	No	Yes	~50%	Bonsignori et al. 68
		Light	K3‐20*01	K1*01	11‐14					
IAVI84	PGT141‐145; PGDM1400‐1412	Heavy	1‐8*01	6*02	18‐27	31‐32	Yes	Yes	6%‐83%	Walker et al. 45; Sok et al. 38
		Light	K‐28*01	K1*01	11‐22					

These mAbs often have substantial breadth, neutralizing 70%‐85% of viruses,^38^, ^46^, ^48^ and in some cases remarkable neutralization potency in the nanomolar range.^37^, ^38^, ^46^, ^47^ The need to understand future vaccine coverage, and more immediately the potential utility of mAbs in passive immunization, has led to studies using large panels of transmitted/founder viruses (the infecting virus that both active or passive immunization strategies will need to block) and assessed which combinations of bNAbs may provide optimal potential clinical benefit.^48^, ^49^ In one such study, a V2‐apex antibody, CAP256‐VRC26.25 was included in both the best 2‐mAb and 3‐mAb combinations, highlighting the potential of this specificity in passive immunization, but also in vaccine‐elicited responses. Similarly, a combination of V2‐directed PGDM1400 with PGT121 (which targets the N332 supersite) has been shown to achieve extraordinary breadth and potency, neutralizing 98% of that particular panel of viruses at a median IC_50_ of 0.007 μg/mL.^38^ Although the breadth of some individual CD4bs mAbs and MPER antibodies exceeds those of V2‐directed broad antibodies, their high levels of SHM and (in the latter case) lipid reactivity and autoreactivity may suggest difficulties in their elicitation by vaccination. In contrast, V2‐apex antibodies may acquire breadth with moderate levels of SHM, often as little as 15% divergent from their unmutated common ancestor (UCA)^47^ (Table 1).

Interest in the V2 region has further increased since the findings of the RV144 vaccine trial, a canary‐pox prime and gp120 protein boost regimen that was tested in Thailand in 2009, and which showed moderate efficacy of 31%.^50^ A subsequent immune correlates analysis showed that vaccine‐induced IgG against V1V2 was inversely correlated with risk of HIV acquisition,^51^ a finding supported by a “sieve analysis” that showed immune pressure in V2 in viruses infecting vaccine recipients^52^ and by several follow‐up studies [reviewed in 53]. However, although such antibodies were able to mediate antibody‐dependent cellular cytotoxicity and virus capture, there is no evidence that the low level protection observed during the RV144 vaccine trial was mediated through neutralization. Nonetheless, RV144 has spawned a wide array of immunologic studies, and follow‐up vaccine trials such as those currently underway in sub‐Saharan Africa in the HVTN 702 trial to test HIV‐1 subtype C tailored equivalents. These trials will undoubtedly continue to reveal insights into the role of non‐neutralizing antibodies to V1V2 and under‐appreciated effector functions of antibodies in HIV vaccine efficacy.

## 3 Structural insights into V2‐apex antibody mediated neutralization

**Figure 3:**
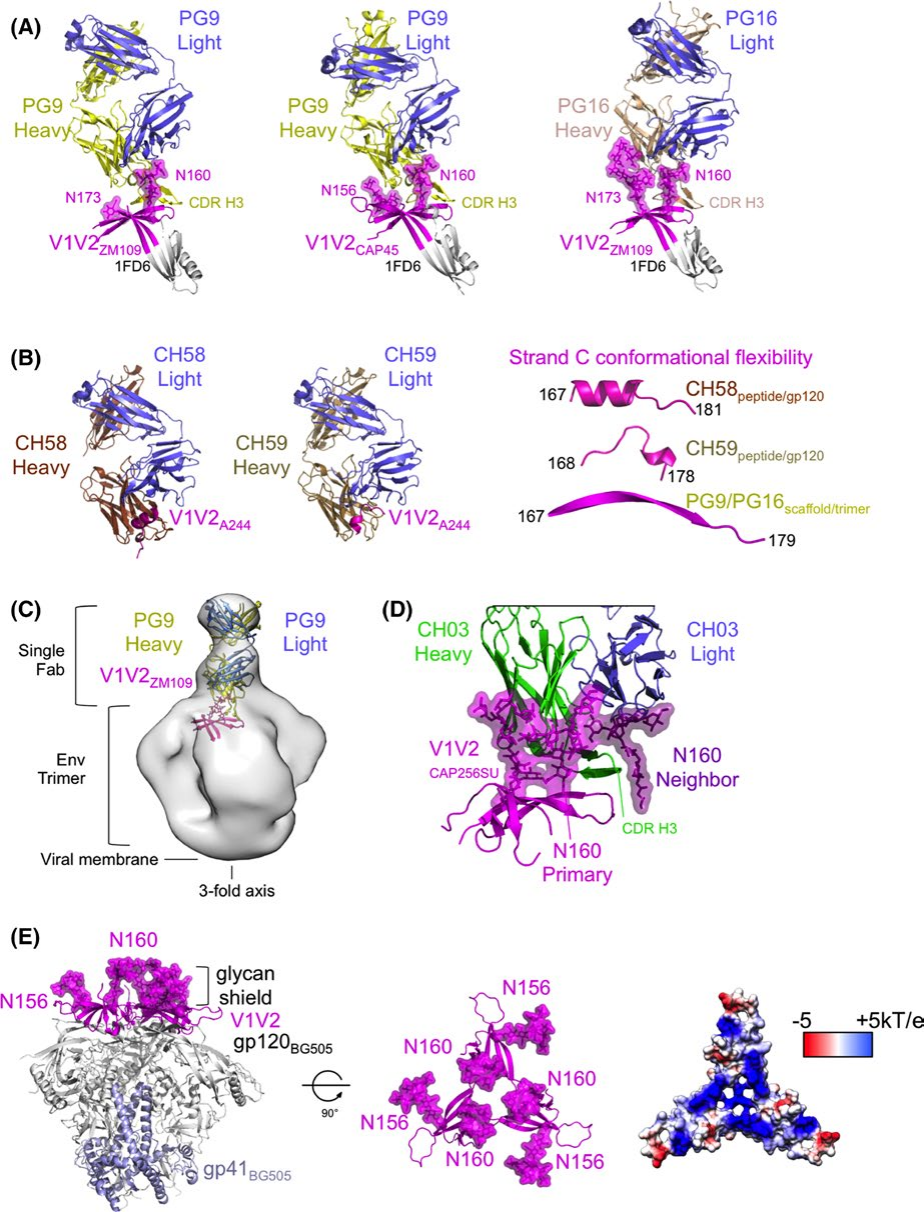
Structural advances in definition of the V1V2 epitope. (A) Crystal structures of monomeric scaffolded‐V1V2 bound by PG9 laid the foundation for the structural definition of the V1V2 domain. PG16 bound to the same scaffold with an alternate glycoform highlighted the specificity of lineage members to heterogeneity in the glycan shield. (B) CH58 and CH59, antibodies isolated from an RV144 vaccine that do not neutralize tier 2 virus, were crystallized with a V2 peptide. These structures, together with Surface Plasmon Resonance data, revealed that the C‐strand of V1V2 adopts multiple conformations on monomeric gp120, masking the broadly neutralized epitope. (C) EM of PG9 in complex with the trimeric BG505 SOSIP.664 together with high‐resolution crystal complexes suggests that the quaternary preference of PG9 is a consequence of binding the N160 glycan of the neighboring protomer. (D) The crystal structure of CH03 bound to a trimeric V1V2 scaffold confirms a strong interaction with the neighboring N160 glycan in addition to the N160 of the primary‐bound protomer. (E) Multiple crystal structures of trimeric Env (SOSIP.664) define the full quaternary epitope of V1V2, which has protective glycans at N156 and N160 and is positively charged

The failure of empirical vaccine approaches for HIV has led to an increased dependence on more structure‐based approaches to immunogen design. Studies of HIV envelope structural biology have provided key insights into the HIV‐1 envelope and broadly neutralizing antibodies, including V2‐directed antibodies, that are able to circumvent its considerable defenses. A crystal structure of the HIV‐1 envelope gp120 core was solved in 1998, and provided the first atomic‐level details of the HIV‐1 Env.^54^ This core construct, however, lacked the V1V2 domain (residues 126‐196, HXB2 numbering), which resisted crystallization for another 13 years. A critical breakthrough in determining the structure of the conformationally variable and highly glycosylated V1V2 domain came through the isolation of glycan‐dependent broadly neutralizing antibodies, such as mAb PG9/PG16 that bound V1V2 only in its native form.^46^ These antibodies led, in 2011, to the first crystal structures of the V1V2 domain, which were achieved using scaffolded constructs, with the V1V2 region engrafted onto a heterologous protein, bound to PG9^55^ (Figure 3A). Importantly, the recently discovered V1V2‐directed bNAbs enabled an “on‐column” method, where mAbs were coupled to the column and used to specifically purify properly folded and glycosylated V1V2 scaffolds. Viral strains ZM109 and CAP45, both clade C viruses, were solved at 1.8 Å and 2.2 Å respectively. The structures not only revealed atomic‐level details regarding the neutralization mechanism of PG9 but also provided the first glimpse at the two variable domains that cap the trimeric viral envelope. V1V2 was shown to comprise a four stranded beta‐sheet with disordered loops between strands A and B and strands C and D, and later structures would reveal an additional strand not seen in the initial scaffolded structures due to dimerization of the V1V2 domains.^56^, ^57^, ^58^ Although, as described above, V1V2 had long been recognized as a highly variable region, the sequence elements comprising the strands of the domain are more conserved than the outer loop regions (Figure 1A). The unusually long CDR H3 engaged strand C of V2 through main‐chain interactions forming a parallel β‐strand along the edge of the V1V2 sheet.^59^ Sulfated tyrosines provided electrostatic side‐chain interactions with this positively charged region buried under the glycan shield. Although the C‐strand of V2 is more conserved than the loop regions, the sequence variations that do exist could be better tolerated due to the CDR H3 main‐chain interactions with the peptide backbone, which is largely sequence‐independent, providing a mechanism for breadth among antibodies targeting this site.

PG9‐bound V1V2 revealed how bNAbs could target this region not only by penetrating the glycan shield, but by using the dense array of glycans, typically regarded as a viral defense, as part of their epitope. PG9 neutralization is dependent upon glycans at residues N156 (or N173 in some strains) and N160, with the latter conserved glycan being critical for recognition (Figure 3A). The scaffolded proteins in the crystal structures were grown in HEK293S GnTI^−^ cells, which lack N‐acetylglucosaminyltransferase I activity, trapping the glycosylation pathway at an early stage. On‐column purification further selected scaffolds with glycosylation profiles favorable to PG9 binding, Man5GlcNAc2 (Man5) glycans at N156 and N160. ZM109 falls among a minority of HIV‐1 strains that do not have a glycan at N156 but instead contains a glycan at residue N173, which is in close three‐dimensional proximity to N156 in the structure and serves as a viable substitute. The co‐crystal structure of PG16 with a scaffolded V1V2 domain from ZM109, grown in cells treated with the α‐mannosidase II inhibitor swainsonine (predicted to induce hybrid‐type glycans), highlighted a difference in glycan specificity for this clonal relative of PG9. These preferences suggest that while glycans at residues N156/N173 and N160 may primarily consist of Man5,^60^ they are not completely homogeneous and that antibody lineages differentiate to accommodate various glycoforms, as has been observed also for glycan‐targeting bNAb specificities to other conserved sites.^61^

Comparison of V2‐apex targeting bNAbs with non‐broadly neutralizing antibodies that target this same region has also yielded useful information. Sieve analysis during the RV144 vaccine trial had detected immune signatures at sites 169 (an important part of bNAb epitopes) and residue 181. This was supported by the isolation of two V2‐directed antibodies, called CH58 and CH59, both isolated from RV144 vaccines. These antibodies, despite apparently targeting conserved elements of V2, did not display broadly neutralizing activity.^62^ Co‐crystal structures of these vaccine‐elicited antibodies with a peptide from the V1V2 domain corresponding to the C‐strand revealed that these antibodies bound to helical and loop conformations considerably different from those seen in the scaffolded structures (Figure 3B). Surface Plasmon Resonance assays with these antibodies and PG9 confirmed that within a population of monomeric gp120s, the C‐strand of V1V2 could adopt multiple conformations, each recognized by a different antibody. These data highlighted the immunologically defensive conformational flexibility of this region, with monomeric gp120, used in the vaccine trial and in ongoing trials, less likely to remain in the conformation recognized by broadly neutralizing antibodies than closed native trimer. Isolation of a similar antibody from a subtype C HIV infected donor suggests that such antibodies are elicited during infection too (C. Van Eeden, L. Morris et al., unpublished data). Isolation of additional non‐broad V2 antibodies after vaccination and infection is likely to provide further information regarding the proportion of V2‐targeting antibodies that have the potential to mature towards breadth.

## 4 Elucidating the trimer structure provides new tools for immunogen design

Broadly neutralizing antibodies that target the V2 region prefer or require a quaternary epitope,^45^, ^46^, ^47^ which has complicated attempts to elicit such antibodies. The establishment of a soluble prefusion closed trimer 63 enabled these antibodies targeting the trimer interface at the apex of the viral spike to be imaged by negative stain electron microscopy (EM). The first structure, a three‐dimensional reconstruction of PG9 in complex with an engineered trimeric envelope called BG505 SOSIP.664, provided a clue as to the quaternary nature of these antibodies.^64^ PG9 bound asymmetrically to the cap of the spike with a stoichiometry of a single Fab per trimer (Figure 3C). Using the threefold symmetry of the Env and fitting the previously determined scaffolded complex crystal structure with PG9, the authors suggested that PG9 likely would interact with the N156 (or N173) and N160 glycans of the bound protomer, but would likely also bind the N160 glycan of a neighboring protomer (Figure 3C). A second EM structure was determined with CAP256‐VRC26.09 bound to a BG505 SOSIP.664 trimer and showed a similar result to that of PG9.^47^ Analysis of a third antibody, PGDM1400, by EM with 2D class averages revealed that this antibody bound closer to the center of the threefold axis than the previous two, suggesting it may bind in a different manner.^38^

Insights into the quaternary requirements for V2‐directed bNAbs and difficulty with crystallizing an asymmetric complex (containing 1 Fab per trimer) led to the screening of trimeric scaffolded V1V2 proteins as tools for crystallization to understand the atomic‐level details of lineages other than PG9/PG16. Through ELISA screening on V1V2 trimeric scaffolds in a 96 well format, a scaffold that bound to V2 bNAbs better than monomeric gp120s was identified. A 3.1 Å structure of CH03 (from donor CH0219) on a trimeric V1V2 scaffold, derived from the virus that superinfected donor CAP256 (described in more detail below), showed a very similar mechanism of engagement to that of PG9^57^ (Figure 3D). Both antibodies bound the C‐strand through main‐chain strand‐strand hydrogen bonds, though PG9 also showed more involved side chain interactions. Both antibodies also bound glycans at N156/N173 and N160. The CH03 structure, however, revealed a glycan interaction not seen in the monomeric structures. Although the orientation of the V1V2 domains in the trimeric scaffold differed from that of the native spike, a glycan from a neighboring protomer, in close proximity to the position of the N160 of a neighboring protomer on the native trimer, bound extensively to CH03 (Figure 3D). This quaternary glycan interaction provided the experimental atomic details to support the hypothesis put forward in the PG9‐BG505 SOSIP.664 EM study described above.^57^ Although an Env‐complexed crystal structure remains elusive for the CAP256‐VRC26 lineage, data from EM, hydrogen‐deuterium exchange, molecular dynamics, and mutagenesis studies all suggest that these antibodies engage the V2 domain through a similar mechanism as PG9/PG16 and CH01‐CH04.^57^ The isolation of these similar formed antibody lineages from multiple donors suggests the existence of an extended class of antibodies.

Since V2‐directed antibodies prefer or require a quaternary epitope, advances in the construction of a soluble trimeric molecule that recapitulates the architecture of the native trimeric viral spike were critical to understanding the nature of the epitope. A crystal structure and high resolution EM structure of BG505 SOSIP.664^64^, ^65^ provided great insight into the details of the epitope. Although the approximate trimeric interface could be calculated from the earlier low‐resolution EM and crystal structures together, the exact details that are critical for immunogen design against this region were not known. Although the resolution limited details of the side chains, the structures confirmed that the scaffolded V1V2 domain seen in complex with PG9 and PG16 recapitulated the overall domain structure in the near‐native closed trimeric conformation (Figure 3E). A higher resolution structure^56^ would later provide greater details, including a fifth strand (C′) not previously seen. The trimeric orientation of V1V2 results in a highly positive region at the apex of the spike that is targeted by the anionic CDR H3 of V2‐directed bNAbs (Figure 3E, right panel). Recent fully glycosylated trimer crystal structures reveal the protective nature of the glycan shield at the apex of the spike.^66^ The extensive glycan coverage requires antibodies targeting the semi‐conserved C‐strand region of V2 to have long CDR H3 in order to penetrate through small crevices to interact with the protein surface. This recent establishment of soluble trimers has thus provided a wealth of information for quaternary epitopes, V2 specifically, and creates an exciting tool for understanding antibody development and vaccine design.

## 5 The antibody precursors and viral triggers of V2‐apex lineages

**Figure 4:**
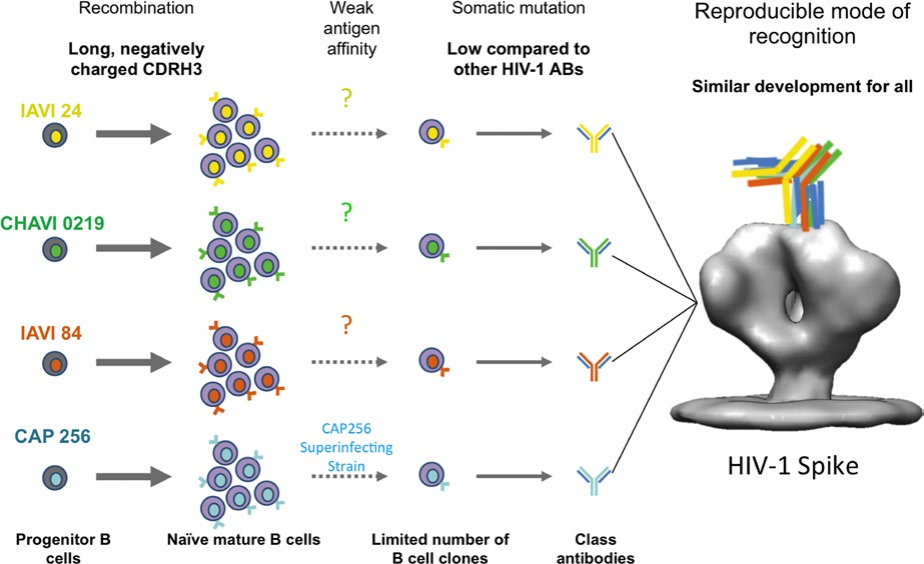
Schematic of B cell ontogeny of the V2‐apex extended class of broadly neutralizing antibodies from four donors. Long, negatively charged CDR H3s are established during immunoglobulin gene recombination, forming progenitor B cells. Unusual viral variants with weak antigen affinity select these rare precursors, which mature to acquire breadth with moderate levels of somatic hypermutation. Mature bNAbs share a common mode of recognition of the HIV‐1 envelope trimer, with many determinants of neutralization present in the unmutated common ancestor. Figure adapted from Zhou et al.^90^

Studies of the ontogeny of other classes of bNAbs have proven to be extremely revealing. In particular the shared germline gene structures and predictable developmental pathways for the CD4 binding site (CD4bs) class of bNAbs have enabled great progress in the development of germline targeting immunogens for this class (reviewed elsewhere in this series). Similar studies have recently been performed for V2‐apex antibodies, which based on commonalities in structure (and ontogeny, described in more detail below) have been proposed to form an “extended class” of antibodies^57^ (Figure 4).

Such studies rely on the comparison of V2‐apex antibodies from multiple donors. The majority of V2 bNAbs were isolated from donors during chronic infection, but despite the absence of early sequences from these antibody lineages, have proved highly informative. For the PG9/PG16 mAbs, little additional lineage information is available and approximations of early members can only be calculated through comparison of the two antibodies and their germline VH/VL genes. In these constructs, the VH or the VH and VJ gene segments are replaced by the corresponding germline genes, however, the CDR H3, a major determinant of neutralization, which spans the V‐D‐J junction, cannot be accurately reverted and is left as the mature form. These germline reverted constructs (referred to as gHgL or reverted unmutated ancestors, RUAs) approximate the properties of a true UCA as well as possible. One version of the reverted PG9 gHgL bound to eight trimeric envelope strains in an ELISA format and neutralized four of 182 strains.^57^ However, PG9 gHgL with one additional amino acid reversion in the CDR H3 failed to induce Ca flux in a B cell activation experiment which included strain ZM233,^67^ one of the four strains shown to be neutralized by the first revertant,^57^ highlighting the importance of accurately determining the CDR H3 for this class of antibody.

Similarly for CH01‐CH04, two versions of a putative precursor (RUA‐1 and RUA‐2) were inferred from the complete sequences of the four lineage members (CH01‐CH04) isolated from donor CH0219 during chronic infection.^68^ They differ by one amino acid in the CDR H3 due to an uncertainty in one non‐synonymous position (G/T at position 401 in the CDR H3) of the calculated ancestor. These variants contain fully reverted VH and VL genes, however, the CDR H3s differ from the most closely related mature lineage members by only one or two amino acids for RUA‐1 and RUA‐2 respectively. These two CH04 early ancestors neutralized four of 24 tier 2 viruses (i.e. neutralization resistant viruses that represent circulating strains) in one study^17^ and RUA‐2 neutralized nine of 201 in a second.^57^ Next generation sequencing (NGS) data of donor CH0219 was later obtained and indicated that the earliest inferred ancestor, which had a maturation rate of only 0.3%, differed by two amino acids in the CDR H3 from the previously defined RUA‐1 or RUA‐2, with one change located at the previously uncertain position.^57^ Notably, these residues (100e and 100f, Kabat numbering) reside in an area of direct contact with V2, although they form hydrogen bonds through main‐chain strand‐strand interactions, which may limit the impact of the side chains in this region.

The same approach was taken with the PGT145/PGDM1400 lineage isolated from donor IAVI84, which differs slightly from the other V2 bNAbs as its CDR H3 is extended at less of an angle and the approach of the antibody to the V2 domain is more centered. Given this, the exact binding mode may differ from the other V2 bNAbs, however, it shares the same genetic characteristics observed in the others. The CDR H3s of most members are 32 residues in length although some, including PGT145, contain a single amino acid deletion. Intradonor phylogenetic analysis of sequences from the mature isolated antibodies as well as NGS data revealed the earliest intermediate that could be well‐defined still had ~6% nucleotide‐level SHM. The CDR H3 had 32 amino acids and a net −5 charge, and did not include sulfated tyrosines, suggesting that many of the determinants of the canonical long, anionic CDR H3 were formed early in the lineage.

Of all the V2 bNAb lineages, only the CAP256‐VRC26 family isolated from a subtype C infected and superinfected donor has been characterized longitudinally from the time of infection, providing key insights that support and extend the studies described above. The earliest cloned antibody, CAP256‐VRC26.01, was isolated at week 59 and 32 other members of the lineage were isolated at weeks 119‐206 by a combination of B cell culture and trimer‐based sorting.^37^, ^47^ NGS was performed at multiple time points to pinpoint when the lineage emerged. At 15 and 30 weeks postinfection, no CAP256‐VRC26 lineage‐related sequences were observed out of ~250,000 sequence reads. However, at week 34 and later time points, hundreds to thousands of lineage sequences were observed.^47^, ^69^ Using the NGS sequences as well as the cloned antibody sequences, a maximum likelihood phylogenetic analysis was used to infer the UCA.^47^ Sequences that differed from the UCA by only a few nucleotides were found in the week 34 and week 38 data, supporting the accuracy of the UCA inference. The reconstructed UCA was able to bind to a trimeric Env spike mimic that bears the V1V2 of the superinfecting transmitted/founder strain CAP256‐SU^57^ and weakly neutralized the superinfecting virus^41^ consistent with the fact that the bNAb response predominantly targeted this viral strain. Thus, the UCA had the key properties of the lineage, with an anionic CDR H3 of 35 amino acids formed in the original VDJ recombination event, rather than a gradual extension by SHM, but required engagement by an unusual viral envelope (see below).

## 6 Triggering precursors of V2‐apex lineages

The long CDR H3s that characterize V2‐apex antibodies range from 24‐37 amino acids (Kabat numbering). These are in contrast with typical antibodies, which have a mean length of 14‐16 amino acids.^70^, ^71^, ^72^, ^73^ Antibodies with such long CDR H3s are relatively rare ‐ those with CDR H3s longer than 24 amino acids comprise only 3.5% of the naïve repertoire while CDR H3s longer than 28 amino acids are only present in 0.43% of native B cells. Notably, CDR H3s longer than 24 amino acids are significantly more common in the naïve repertoire than in mature B cells (in both the IgG and IgM memory subset), possibly due to autoreactivity and deletion at checkpoints,^74^, ^75^ posing an additional challenge to eliciting such responses. Antibodies with long CDR H3s tend to be enriched for the use of a subset of D and J gene segments,^75^ which are associated with conserved sequence motifs. These motifs include the tyrosine‐sulfated YYD motif seen in both PG9/16 and CAP256‐VRC26 lineage members that contributes to the anionic nature of the CDR H3, though its precise role in epitope recognition differs between the two antibodies.^57^ The use of two tandem D genes, termed D‐D fusion, may also contribute to long CDR H3s,^76^ although this unlikely to be the source of the ultra‐long CAP256‐VRC26 CDR H3.^47^ Despite these low frequencies, in the context of between 10^10^ and 10^11^ B cells in vivo, it is likely that as for other rare bNAb precursors,^77^ sufficient numbers of V2 apex precursors exist in the naïve repertoire to be reliably recruited by an efficient germline‐targeting immunogen.

Studying the neutralization and envelope binding profiles of V2‐apex antibody precursors may provide clues as to the nature of the bNAb‐initiating virus that triggered these lineages. Overall, studies of CAP256 and of other lineages suggest that V2‐apex UCAs are unusual clones selected from the naïve repertoire, which do not neutralize or bind the vast majority of strains. Indeed, in CAP256 for which the most accurate V2‐apex precursor has been determined, the UCA neutralized only the superinfecting transmitted/founder virus, but not the primary transmitted/founder virus or any other virus from a panel of two hundred.^69^ Identifying the virus that triggered the lineage was therefore a major interest, especially given that the CAP256‐VRC26 lineage was detected only 20 weeks after superinfection.^47^, ^69^ NGS of the viral V2 region revealed variability by week 34 (the first time point at which CAP256‐VRC26 transcripts were detected) with descendants of both the primary and superinfecting viruses and recombinants thereof all represented. Three Env variants isolated from week 34 and derived from the superinfecting virus were more sensitive to neutralization by the UCA than the parental CAP256‐SU virus, suggesting that they or similar bNAb‐initiating envelopes engaged the B cell bearing the UCA, thereby triggering the lineage.^69^

Identifying additional such V2‐apex bNAb‐initiating envelopes has potential utility for immunogen design, but relies on detailed longitudinal sampling, and on these envelopes being generally more reactive with multi‐donor V2‐apex precursors than other viral envelopes. Previous studies of V2‐apex antibodies had sought to identify germline‐reactive envelopes for individual lineages as the basis of future immunogens.^68^ To empirically determine strains that may inherently offer the most promising combinations of attributes sought in a V2 immunogen, large panel neutralization assays were carried out with mature, intermediate and germline reverted V2 bNAb antibodies from all four known donors. Where longitudinal data were available, UCAs were used, and otherwise V‐gene reverted variants with mature CDR H3s served as approximate earliest ancestors.^57^, ^68^, ^78^ Notably, only a small number of stains were neutralized by these V2 bNAb precursors, with several of the strains such as WITO.33 and ZM233 overlapping between V2‐bNAb donors.^57^ A parallel independent study also identified some of the same strains using a similar approach.^79^ Although each strain individually showed some traits to set them apart from the consensus, no universal particular elements could be detected among the sensitive strains. Interestingly, one of these strains included the CAP256 superinfecting virus (CAP256‐SU), a variant of which elicited the CAP256‐VRC26 lineage,^69^ highlighting the potential utility of longitudinal studies of V2‐apex donors in identifying additional useful viral strains for inclusion into immunogens described in more detail below.

## 7 Maturation of breadth from bNAb precursors

Beyond the initial engagement of precursors with long CDR H3, a key recent focus has been on how V2‐apex antibodies (and other bNAb specificities) mature to acquire breadth. Longitudinal analyses of both antibody and virus during maturation of the CAP256‐VRC26 lineage have highlighted the substantial role of viral diversification in the emergence of neutralization breadth.^47^, ^69^ Phylogenetic analysis of the CAP256‐VRC26 lineage shows two main branches. The first branch is thought to be an evolutionary dead end, in that members of the branch could not be detected among B cell transcripts from time points after week 119. In contrast, the second sublineage continued to evolve, with sequences observed at all time points up to 206 weeks postinfection. Affinity maturation that enabled progressively better binding and neutralization was largely in the CDR H3.^47^ This included a signature sequence that emerged early in the development of the second branch: a cysteine‐cysteine disulfide bond in the CDR H3, along with an arginine at the base of the CDR H3, which may have stabilized the conformation of the CDR H3 and enhanced neutralization breadth and potency.^47^

**Figure 5:**
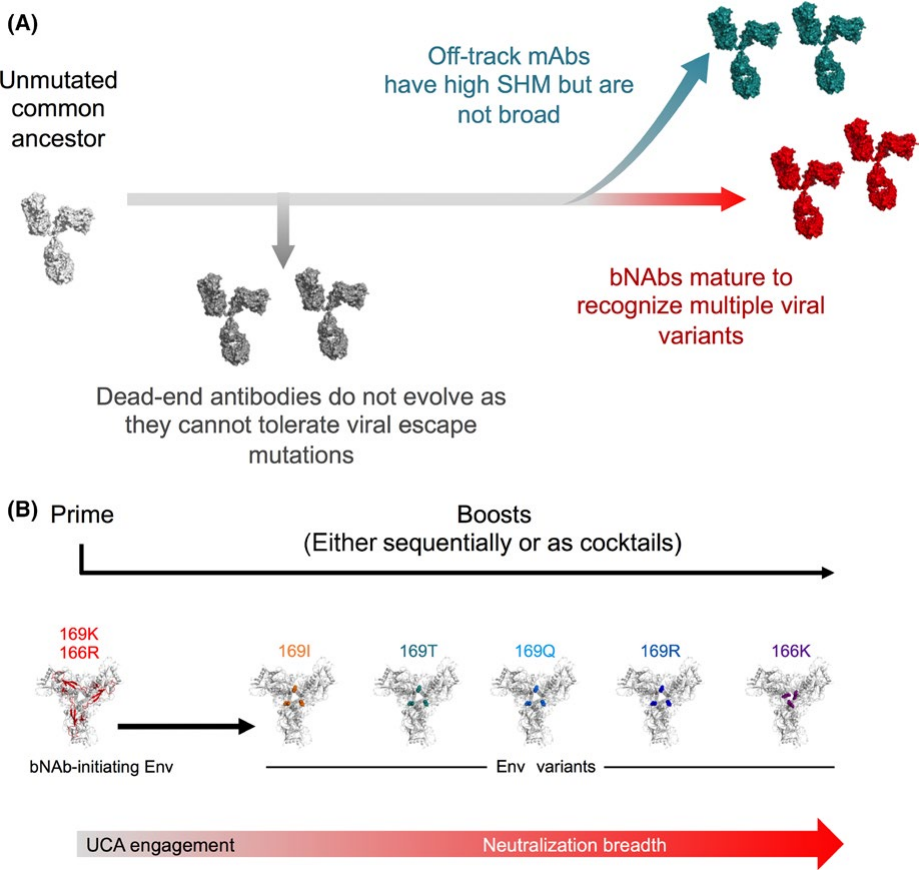
Antibody maturation pathways and their implications for HIV vaccine design. (A) Schematic depicting how CAP256 viral evolution results in the engagement of the CAP256‐VRC26 UCA (light gray) and the subsequent development of two distinct mAb sublineages. The sublineage with restricted evolution contains dead‐end mAbs (dark gray), which cannot tolerate viral escape mutations. The continually evolving sublineage contains bNAbs (red) and off‐track mAbs (turquoise). Although both bNAbs and off‐track mAbs have high levels of SHM, only the former mature to recognize multiple immunotypes, and thus acquire breadth. Adapted from 69 (B) Schematic depicting a vaccine approach that seeks to recapitulate key viral events that drove neutralization breadth in the CAP256‐VRC26 lineage by the use of sequential or cocktail approaches. Adapted from 69

Comparison of the neutralization capacity of mAbs isolated from both branches against longitudinally sampled autologous viruses provided a mechanism for these distinct evolutionary outcomes. The antibodies in the “dead‐end” branch were unable to tolerate viral escape mutations that were selected as the viral quasispecies evolved to evade the early bNAb responses^69^ and were therefore no longer selected for. Such dead‐end antibodies may be the reason for the rapid loss of lineage transcripts observed for several bNAb lineages.^80^ In contrast, within the second branch, increasing breadth of mAbs was shown to be correlated with a capacity to neutralize viral escape variants (termed “immunotypes”) at positions 166 and 169 in the C‐strand suggesting that continued adaptation to emerging viral variants contributed to the development of breadth.^69^ Similar findings and viral escape pathways have been observed for another V2‐apex donor for whom longitudinal data are available (E. Landais and P. Poignard, unpublished). Interestingly, within this continually evolving CAP256 sublineage, antibodies with both broad and narrow neutralization breadth were intermingled within the phylogenetic tree. This clustering of broad and narrow lineage members suggests that individual amino acid changes, rather than overall sequence identity, have large effects on neutralization capacity. Indeed, although many of the broader members of the lineage had high levels of affinity maturation, equally mutated but strain‐specific antibodies, termed “off‐track antibodies” were also detected in the lineage (Figure 5A), with similar observations reported for the PGT145/PGDM lineage and for bNAb lineages targeting other epitopes.^38^, ^45^, ^80^, ^81^ These observations suggest that somatic antibody variants with a variety of neutralization capacities frequently evolve side‐by‐side during chronic HIV infection (Figure 5A). This evolution is likely to be driven by the concurrent diversification of the viral quasispecies: while some antibody mutations may improve the binding to specific autologous viral variants encountered in the germinal center, these may not be the same mutations that improve neutralization breadth against heterologous viruses (which the antibodies never encounter). For example, some off‐track antibodies in the CAP256‐VRC26 lineage appear to target an immunotype among the circulating autologous viral quasispecies that is rare in globally circulating viral strains, likely accounting for the strain‐specificity of that antibody (D. Sacks, P.L. Moore et al., unpublished).

These studies of bNAb maturation in donor CAP256 have defined a mechanism for how accumulating viral variants contributed to V2‐apex bNAb development. Viral escape from early members of the antibody lineage creates multiple viral immunotypes. In parallel, SHM generates antibodies with differential abilities to engage these epitope variants. Those antibody sublineages that are able to tolerate variability at key epitope contacts may develop breadth. These findings have implications for ontogeny‐based immunogen design, suggesting that breadth may require the incorporation of multiple immunotypes into vaccines, to drive antibody maturation toward tolerance of diversity^69^ (Figure 5B). Ideally, B cell‐lineage based vaccine strategies would aim to drive maturation of bNAbs, while limiting the loss of B cells that are unable to tolerate immunogen diversity and also avoiding off‐track antibodies. Realistically, the stochastic nature of antibody maturation is likely to make this impossible. However, in the context of infection, dead‐end and off‐track antibodies in many donors do not appear to hinder the development of breadth, a scenario supported by modeling of germinal center reactions in the context of cocktails of immunogens (J.S. Shaffer and A.K. Chakraborty, unpublished).

## 8 Incorporating ontogeny into structure‐based immunogen design

Studies of the ontogeny of V2‐apex antibodies have led to increased efforts to incorporate structural and virological findings into targeted immunogens. Many clinical trials based on immunization with monomeric gp120 have failed to elicit bNAbs, including those directed to the V2‐apex.^82^ The structural explanation for this latter finding is that such gp120 monomers do not form the full V2‐apex epitope and can adopt multiple conformations of C strand.^62^ Scaffolded V1V2 designs offer immunogens that are advantageous in their ability to focus the immune response to a specific domain. However, there are a number of limitations for these constructs. Many are overexpressed in cells where the glycosylation machinery becomes overwhelmed, resulting in heterogeneously glycosylated sites. These results are evident from ladders of differentially glycosylated scaffold bands on an SDS PAGE gel.^55^ Additionally, a substantial proportion of scaffolds do not fold properly. These two issues could be overcome through on‐column purification methods, where misfolded or improperly glycosylated molecules flow past the antibody and only those that are suitable immunogens are bound, resulting in a homogeneous population of antibody‐bound scaffolds. If these scaffolds could be removed from the bound antibody without disturbing the scaffolded structure then they could be used as monomeric immunogens. However, the remaining, and possibly most important, limitation is that no published scaffolds correctly recapitulate the full trimeric orientation of a properly folded native trimer.

The advance of soluble trimers such as BG505 SOSIP.664 has overcome many of the obstacles associated with monomeric immunogens and therefore marks a critical and exciting advance toward targeting V2‐directed bNAbs. The quaternary nature of V2‐directed bNAbs elicited through natural infection strongly suggest that effective immunogens targeting this ontogeny need to present the full epitope present on the native viral spike. Availability of this soluble trimer has provided a great deal of structural information (described above) recently enabling the optimization of immunogen design targeting specific epitopes, particularly those of a quaternary nature. Atomic‐level details from multiple structural studies of this construct has facilitated additional stabilizing mutations such as a disulfide between residues 201 and 433 that prevents the trimer from conformationally shifting upon binding of CD4. However, providing a stabilized architecture of the trimeric V1V2 epitope may not be sufficient to elicit a response. Effective immunogens must also take into account the distance and orientations of each V1V2 domain relative to each other, the equilibrium of the domain at the cap (the V1V2 domain may shift as observed by smFRET^83^), stabilization of the C‐strand itself, exact residue requirements, and the appropriate glycosylation. Without the right strain there is very little chance of engagement even if the appropriate antibody precursor is present. Critically, as mentioned above, precursors to V2 bNAbs do not neutralize or bind the vast majority of naïve strains.^57^, ^67^, ^68^, ^69^, ^79^ This suggests that, even if the precursors exist in a naive repertoire and a soluble trimer that perfectly recapitulates the native spike is present, V2 bNAb precursors are unlikely to be stimulated unless the appropriate strains are used.

The identification of a few strains that have a higher probability of engaging V2‐apex bNAb precursors from multiple donors, described above,^57^, ^79^ opens up possibilities for incorporating these into trimeric immunogens. However, the use of these viral strains to elicit V2‐apex bNAbs still includes a number of obstacles. The SOSIP mutations that are used to stabilize soluble trimers such as BG505 SOSIP.664, have been now been integrated into a small number of additional strains to form soluble trimers,^66^, ^84^, ^85^, ^86^ and similarly a few strains form stable trimers with a set of mutations termed NFL.^87^ However, most strains are unstable or do not display full cleavage,^63^, ^84^ thereby resulting in trimers with heterogeneous protomers which can unfavorably alter their antigenic profile. Several solutions are currently available such as replacing the V1V2 domain (residues 126‐196) of the BG505 SOSIP.664 with those of the strain of interest.^57^ This strategy requires that the V3‐V1V2 interactions be compatible between strains. Alternatively, the entire gp120 may be replaced while maintaining core residues of BG505 in the N and C termini, as well as gp41 (M.G. Joyce et al., unpublished). One attractive alternative is to remove the cleavage site altogether: this can be accomplished through placing a flexible peptide linker in its place^88^, ^89^ although this method requires additional stabilization of other regions. This method does occlude a target site of vulnerability in the fusion peptide,^41^ however, if the goal is to target V1V2 bNAbs specifically, this is not a critical epitope to retain. The great advantage of this method as a vaccine strategy is that it removes the requirement for furin co‐expression, which simplifies the manufacturing pathway for stable soluble protein. This provides a means of delivering optimized cleavage‐independent trimers, which could easily be engineered to incorporate the multiple immunotypes required for the development of breadth during natural infection, via genetic immunizations.

## 9 Conclusions

With the availability of a soluble trimer that mimics that native spike, a great many ideas for immunogen design that were technologically unavailable just a few years ago are now possible. An explosion of crystal and EM structures^56^, ^57^, ^64^, ^65^, ^66^ has also provided atomic‐level details to further stabilize the molecules. Replacement of the V1V2 domain or design of gp120 chimeras which require an understanding of the atomic‐level details of the trimeric interactions are possible now that structures of the trimers are available. Recent advances have also been achieved in structurally defining the glycan shield surrounding the trimer that must be accounted for by V2 bNAbs. Additionally, technology to assemble these trimers onto nanoparticles is still under development but the field is moving forward at an extraordinary pace with these new tools. Open questions still remain regarding the exact mechanism of how specific strains are engaged by V2 bNAb precursors, what the optimal glycan requirements are for eliciting bNAbs and whether vaccine strategies need to incorporate diversity (and if so how much) to drive antibodies toward breadth, as suggested by studies of infection. However, stabilized trimers presenting the appropriate quaternary epitope for stimulating V2 bNAb precursors represent one of the most exciting immunogen strategies, and has only become possible with the through our recent understanding of these antibody ontogenies as well as the emergence of sophisticated soluble trimeric reagents in the past few years.
